# IL-23 Promotes γδT Cell Activity in Dry Eye Disease Progression

**DOI:** 10.1167/iovs.66.2.10

**Published:** 2025-02-04

**Authors:** Yanxiao Li, Zan Luo, Zihao Liu, Xinhao Zhu, Peter S. Reinach, Ling Li, Wei Chen

**Affiliations:** 1 State Key Laboratory of Ophthalmology, Optometry and Visual Science, Eye Hospital, Wenzhou Medical University, Wenzhou, China; 2 National Clinical Research Center for Ocular Diseases, Eye Hospital, Wenzhou Medical University, Wenzhou, China; 3 Ningbo Eye Institute, Ningbo Eye Hospital, Wenzhou Medical University, Ningbo, China

**Keywords:** γδT, IL-23, dry eye

## Abstract

**Purpose:**

Conjunctival-resident γδT cells, the predominant ocular source of interleukin-17A (IL-17A), play crucial roles in dry eye disease (DED) pathogenesis. The upstream regulators of these cells are unknown. This study evaluated the role of conjunctival IL-23 expression in mediating γδT cell generation and elucidated its contribution to dry eye inflammatory responses.

**Methods:**

Single-cell RNA sequencing (scRNA-seq) was used to identify and quantify conjunctival mRNA molecules in γδT cells in mice. The IL-23 level increased in wild-type (WT) and decreased in γδT-deficient (TCR*δ*^–/–^) mice after dry eye was induced via an intelligently controlled environmental system (ICES). Flow cytometry and transcriptome sequencing were used to investigate the impact of the changes in IL-23 expression on human γδT cells.

**Results:**

The expression of the IL-23 receptor (IL-23R) was greater in γδT cells than in other conjunctival cell types, such as CD4+ T cells, CD8+ T cells and epithelial cells. An increase in IL-23 led to an increase in γδT cell density, which was proportional to dry eye severity. However, in the TCR*δ*^–/–^ mice, the upregulation of IL-23 failed to increase the expression level of IL-17A and the severity of dry eye. Furthermore, increases in the expression of IL-23 and the number of γδT cells were evident in the ocular surface cells of patients who developed visual display terminal syndrome.

**Conclusions:**

An increase in conjunctival IL-23 expression contributes to the induction of the DED inflammatory response through interactions with its cognate receptor on γδT cells and the promotion of their proliferation. The findings of this study suggest that the suppression of IL-17A through the blockade of IL-23R activation may be a viable target for improving the management of inflammation in DED patients.

Dry eye disease is a widespread multifactorial disease that can result from immune-mediated damage.^1^ Various factors contribute to this condition, including inflammation-induced tissue injury,^1^ environmental stressors,^2^ and hyperosmotic damage.^3^ Prolonged screen exposure, low humidity, and air pollution exacerbate ocular surface irritation, disrupting tear film homeostasis.^4^^–^^6^ This disturbance triggers inflammatory responses, promoting the release of proinflammatory cytokines and immune cell infiltration, thereby worsening tissue injury.^7^^,^^8^ The subsequent loss of epithelial integrity and goblet cell function further impairs tear production and stability, perpetuating a cycle of inflammation and dryness.^9^^,^^10^ As the ocular surface becomes unstable, symptoms such as burning and itching manifest, significantly affecting quality of life.^11^^–^^13^ The underlying pathophysiological changes include a loss of tear film homeostasis and the development of inflammation that leads to visual impairment.^14^^,^^15^ The Tear Film & Ocular Surface Society Dry Eye Workshop II asserts that the core mechanism of DED is ocular surface inflammation, with IL-17A being a key cytokine.^16^ Studies have shown increased expression of IL-17A in both animal models of dry eye and the ocular surface of patients with DED.^17^^–^^20^

Historically, it was believed that IL-17A was predominantly produced by Th17 cells. In 2009, Chauhan et al.^21^ discovered the presence of IL-17R on the cornea and conjunctiva, with PCR analysis also revealing the increased expression of IL-17A mRNA; however, the specific cellular source of IL-17A on the ocular surface was not directly examined in that study. Instead, they investigated the relationship between cervical lymph node Th17 cells and IL-17A.^21^ In 2017, Zhang et al.^22^ used immunohistochemistry to observe an increase in CD4+ cell infiltration in the corneal epithelium and detected increased IL-17A mRNA expression in the cornea in DED; however, the origin of IL-17A in the cornea was not directly traced to CD4+ cells. Other research has revealed that the main source of IL-17A production in the conjunctiva is γδT cells, with Th17 cells contributing to a lesser extent. Ablation of γδT cells by antibody depletion or genetic deletion of TCRδ alleviates ocular surface damage in murine DED.^23^^,^^24^ Therefore exploring the signaling mediators that induce γδT-cell activation is important for the future treatment of DED.

The mediators that control γδT cell expression and function are known to exist in other tissues. The interaction of IL-17A with γδT cells increases proinflammatory IL-17A expression, which in turn triggers psoriasis onset, as well as its progression.^25^^–^^27^ Dysregulation and upregulation of IL-23 activate Th17 cells^28^; this response induces increases in proinflammatory cytokine expression levels, which contributes to tissue inflammation and necrosis.^28^^–^^31^ IL-23 expression is an essential modulator of cytokine activation and expression by various immune cell types in colitis,^32^ including natural killer (NK) cells, intraepithelial lymphocytes, innate lymphoid cells and Th17 cells.^32^^–^^36^ IL-17A promotes the suppressive ability of myeloid-derived suppressor cells (MDSCs), which are a heterogeneous population of immature myeloid cells that have the ability to suppress immune responses, thereby facilitating tumor evasion.^37^ Additionally, MDSCs can induce IL-17A expression through interactions with the IL-17A and IL-23 signaling pathways in γδT cells.^37^

The γδT cell function is increased by many different mediators in numerous diseases and tissues. In contact hypersensitivity, dendritic epidermal T cells, a unique subset of γδT cells in the skin epidermis, are activated and in turn secrete IL-17 in an IL-1β–dependent manner.^38^ Similarly, in liver fibrosis, IL-1β activates mTORC2 signaling, which upregulates CXCR3 and enhances the secretion of IFN-γ by γδT cells, mitigating inflammation at the site.^39^^,^^40^ A distinct group of γδT cells characterized by CXCR3+ CXCR6+ expression, which are found predominantly in the mouse liver, exhibit high IL-2R expression levels and display tissue residency traits from the embryonic stage through adulthood.^41^ IL-7 has been identified as a major promoter of γδT cell thymic development, maturation, differentiation into peripheral organs, and maintenance of cellular homeostasis.^42^^–^^46^ Upon stimulation with IL-12 and IL-18 in vitro, Eomes+ γδT cells exhibit increased rates of proliferation and IFN-γ secretion.^47^^–^^49^

We showed that there is appreciable IL-23R expression on γδT cells in the mouse conjunctiva. Diminishing IL-23 levels reduces ocular surface inflammation in mice. Importantly, increases in IL-17A release occur in WT mice but are absent in TCR*δ*-deficient mice, which lack γδT expression, along with a decrease in ocular surface inflammation. This dampening of IL-23–induced inflammation in TCR*δ*-deficient mice indicates that the increase in ocular surface inflammation in WT mice is dependent primarily on the interaction of IL-23 with its cognate receptor on γδT cells rather than other conjunctival cells. We further validated the commonality of the IL-23/γδT immune inflammation signaling pathway axis in humans and mice. Overall, our findings elucidate the role of IL-23 as an upstream regulator of γδT cells, which exacerbates the severity of DED through increases in IL-17A expression.

## Material and Methods

### Clinical Subjects

The Institutional Review Board of Wenzhou Medical University approved all procedures involving human subjects in strict adherence to the Declaration of Helsinki (Ethics Approval Number: 2022-132-K-101-01). Prior to their inclusion in the study, all participants provided written informed consent. The inclusion criteria for participants were as follows: healthy individuals aged between 18 and 40 years, with no abnormalities in the anterior or posterior segment of the eye confirmed by slit-lamp examination, and a reported daily visual display terminal (VDT) usage time exceeding four hours. Exclusion criteria were (1) a history of ocular trauma or surgery, (2) current contact lens use, (3) use of ocular medications, (4) autoimmune disorders or immunosuppressive drug use, (5) any ocular diseases other than dry eye and refractive errors, (6) participation in any clinical trial in the last 30 days, (7) inability to complete required procedures or follow-ups, and (8) assessed as unsuitable for participation because of health conditions or other factors including monocular vision impairment, low vision or blindness, reading disorders, severe trichiasis leading to corneal epithelial defects, or severe adhesions after double eyelid surgery. Before baseline assessment, participants were required to maintain a rest state for at least seven days, limiting daily VDT usage less than three hours to minimize the impact of prior long-term VDT exposure. Subjects were required to engage in visual terminal usage for more than eight hours per day,^2^^,^^50^ over a minimum period of one week. Additionally, ocular samples were obtained at both baseline and week 1 for flow cytometry (FCM) and RNA-sequencing analyses. To monitor visual terminal usage, we used the “Digital Wellbeing” features available on Android devices and the “Screen Time” feature on iOS devices, both of which provide detailed hourly usage statistics. For computer monitoring, we used Tockler, a software that supports Windows, Mac, and Linux, to track computer usage time (Supplementary Fig. S1A). These tools enabled us to collect comprehensive data on the visual terminal usage of our subjects. The sample size was evaluated using G*power 3.1 software (University of Dusseldorf, Dusseldorf, Germany). We calculated based on repeated measures ANOVA, considering the single-group repeated measures design of our study. On the basis of previous clinical researches,^51^^–^^53^ we moderately set the effect size (f) of VDT, α and power (1-β) to 0.3, 0.05 and 0.80, respectively. Finally, a sample size of 24 participants would be sufficient according to the calculation results, and we included a total of 26 participants to ensure adequate statistical power of our study. The specific process of the clinical trial is shown in Supplementary Figure S1B.

### Animals

C57BL/6J (B6 WT) mice, aged six to eight weeks, were obtained from Shanghai Jiesijie Experimental Animal Co. Ltd. TCR*δ*^–/–^mice on the C57BL/6J background were procured from The Jackson Laboratory (Bar Harbor, ME, USA; Jackson Stock No: 002120). All animal experiments adhered to the ARVO Statement for the Use of Animals in Ophthalmic and Vision Research, with ethics approval number wydw2023-0027.

### DED Induction

To induce DED, adult female mice were housed in an ICES that rigorously controlled the relative humidity setting between 15.0% and 20.0%, airflow at 2.2 ± 0.2 m/s, and temperature maintained at 22°C ± 2°C. Conversely, the control group was composed of mice that were age- and sex-matched to the experimental subjects and housed under standard conditions with a relative humidity of 60% to 80%, an absence of airflow, and a stable temperature range of 21°C to 23°C.^54^ On the sixth day of the experiment, the mice were euthanized for the collection of conjunctival tissue.

### Preparation of Single-Cell Suspension For FCM

Conjunctival tissues were harvested by excising both the palpebral and bulbar conjunctiva. After excision, the samples were immediately rinsed and agitated in PBS supplemented with 20 mM EDTA (Beyotime, Shanghai) and maintained at 37°C for 15 minutes to facilitate tissue preparation. The tissues were subsequently diced into smaller fragments and enzymatically digested in RPMI 1640 medium (Thermo Fisher Scientific, Waltham, MA, USA) enriched with collagenase IV (provided by Gibco, Thermo Fisher Scientific; at a concentration of 1000 U/mL) at 37°C for 30 minutes. For the collection of ocular surface cell samples, swabs (Biocomma, Shenzhen) were used; each swab was meticulously processed to dissociate the collected cells into RPMI 1640 medium. The cellular samples obtained via this method were then prepared for analysis via FCM.

### Flow Cytometry Analysis 

For cell-surface staining, the cells were blocked with FcR block (BioLegend, San Diego, CA, USA) for five minutes and stained with a fluorescent dye-conjugated mAb for 30 minutes at 4°C. For intracellular cytokine staining, the cells were stimulated for four hours with PMA (50 ng/mL; Sigma‒Aldrich, St. Louis, MO, USA) and ionomycin calcium salt (500 ng/mL; Sigma‒Aldrich) in the presence of brefeldin A (2 µg/mL; Sigma‒Aldrich) for two hours. The cells were stained with surface markers for 30 minutes, fixed and permeabilized with commercial kits (Foxp3/Transcription Factor Staining Buffer; eBioscience, San Diego, CA, USA), and stained for cytokines for 45 minutes at 4°C. Unless otherwise indicated, all of the antibodies were obtained from BD Bioscience (San Jose, CA, USA). The antibodies used for flow cytometry analysis staining were APC/Cy7-anti-mouse-CD45 (clone 30-F11), PE-anti-mouse-CD3 (145-2C11), BV785-anti-mouse-CD4 (RM4-5; BioLegend, San Diego, CA), PerCP/Cy5.5-anti-mouse-CD8 (53-6.7), BV421-anti-mouse-TCRγδ (GL3), BV711-anti-mouse-IL-23R (O78-1208), AF647-anti-mouse-IL-17A (TC11-18H10), APC/H7-anti-human-CD45 (2D1), and PE-anti-human-TCRγδ (B1). FCM data were collected via a Cytoflex flow cytometer (Beckman Coulter, Inc, Southfield, MI, USA) or an Attune NxT V6 flow cytometer (Thermo Fisher Scientific) and analyzed with FlowJo software V10 (Tree Star Inc, Ashland, OR, USA).

### Corneal Fluorescein Staining

To evaluate the integrity of the corneal epithelium at baseline and on day 6, a 5% fluorescein solution was applied. Specifically, 0.5 µL of the solution was instilled into the lower conjunctival sac using a pipette gun. After a waiting period of three minutes, corneal epithelial staining was meticulously graded under a slit-lamp microscope, and a cobalt blue filter was used to enhance visualization. The assessment of punctate staining was conducted in a blinded manner, using a standardized grading scale provided by the National Eye Institute (Bethesda, MD, USA), which ranges from 0 to 3. This scale was applied to evaluate the staining intensity across five specific regions of the cornea: central, superior, inferior, nasal, and temporal.

### Reverse Transcription Quantitative Polymerase Chain Reaction

Conjunctival tissues were excised from the mice, and total RNA was extracted using an RNeasy Mini Kit (Qiagen, Crawley, UK) following the manufacturer's protocol. The RNA concentration was determined by measuring the absorbance at 260 nm, and the RNA was subsequently stored at −80°C. Complementary DNA (cDNA) was synthesized from 0.5 µg of total RNA using random primers and M-MLV reverse transcriptase (Applied Biosystems, Paisley, UK). The sequences of the primers used were as follows: for glyceraldehyde-3-phosphate dehydrogenase, sense, 5′-ATGTTCGTCATGGGTGTGAA-3′, and antisense, 5′-GGTGCTAAGCAGTTGGTGGT-3′; for IL-1R1, sense, 5′- GTGCTACTGGGGCTCATTTGT -3′, and antisense, 5′- GGAGTAAGAGGACACTTGCGAAT -3′; for IL-1β, sense, 5′-ATGATGGCTTATTACAGTGGCAA-3′, and antisense, 5′-GTCGGAGATTCGTAGCTGGA-3′; for IL-18, sense, 5′- GACTCTTGCGTCAACTTCAAGG-3′, and antisense, 5′- CAGGCTGTCTTTTGTCAACGA -3′; for IL-18R1, sense, 5′- TCACCGATCACAAATTCATGTGG -3′, and antisense, 5′- TGGTGGCTGTTTCATTCCTGT -3′; for IL-23R, sense, 5′- TTCAGATGGGCATGAATGTTTCT-3′, and antisense, 5′- CCAAATCCGAGCTGTTGTTCTAT-3′; for IL-23, sense, 5′-ATGCTGGATTGCAGAGCAGTA-3′, and antisense, 5′-ACGGGGCACATTATTTTTAGTCT-3′. Quantitative PCR analysis was performed using the Power SYBR Green PCR Master Mix (Applied Biosystems) and an Applied Biosystems Quant Studio 6 Real-Time PCR System (Applied Biosystems). The relative expression levels of the target mRNAs were normalized to those of glyceraldehyde-3-phosphate dehydrogenase as an internal control.

### Immunofluorescence

Conjunctival tissues were excised and immediately embedded in optimal cutting temperature (OCT) compound (Sakura Finetek USA, Inc., Torrance, CA, USA), followed by rapid freezing in liquid nitrogen. For fixation, the sections were incubated at room temperature in 4% paraformaldehyde for 15 minutes, followed by three washes with 1× PBS. Permeabilization and blocking were achieved by incubating the sections in 0.4% Triton X-100 blocking buffer at room temperature for one hour. Primary antibodies diluted 1:200 (TCR γ/δ antibody [Santa Cruz Biotechnology, Dallas, MA, USA]; anti-IL23R antibody [Abcam, Cambridge, UK]; TCR gamma/delta antibody [Novus, Centennial, MA, USA]) were applied overnight in blocking buffer. After being washed three times with PBS, the sections were incubated for two hours with secondary antibodies conjugated to Alexa Fluor 488 and 594 diluted 1:200 (anti-rabbit IgG Alexa 488 and anti-hamster IgG Alexa 594; Invitrogen, Carlsbad, MA, USA). After three washes with PBS, the slides were mounted with anti-fade mounting medium containing DAPI (Beyotime Institute of Biotechnology, Jiangsu, China), sealed with clear nail polish to prevent drying, and stored at 4°C. Visualization was performed using a laser scanning confocal microscope (LSM 880; Carl Zeiss AG, Oberkochen, Germany).

### Whole-Mount Immunofluorescence Staining of the Human Conjunctiva

Conjunctival tissues were obtained from three organ donors from the Eye Bank of Wenzhou Medical University. The donors, aged 20–56 years, included one female and two males of Han ethnicity, who explicitly consented to donate their tissues for clinical usage or scientific research by signing consent forms. The cause of death was either brain death or cardiopulmonary arrest. Tissues were collected less than six hours postmortem, placed in the corneal storage solution Optisol-GS (Bausch & Lomb, Rochester, MA, USA), and processed within 24 hours. After the fascia and fat were removed, the tissues were fixed overnight in 4% paraformaldehyde, permeabilized for 48 hours in a solution containing Triton X-100, and then incubated with primary antibodies (TCR γ/δ antibody [Santa Cruz Biotechnology, Dallas, TX, USA]; anti-IL23R antibody [Abcam, Cambridge, UK]) at a dilution of 1:200. The secondary antibodies were applied for another day at a dilution of 1:500 after thorough washing with PBS. Nuclei were stained, and the samples were mounted with glycerol-based mounting medium. Observations and image capture were performed under a fluorescence microscope.

### Periodic Acid-Schiff Staining of Goblet Cells

After removal, the eyeballs and eyelids were fixed in a solution comprising 10% formaldehyde, 40% absolute ethanol, 40% water, and 10% glacial acetic acid for 12 to 24 hours at room temperature and then embedded in paraffin. Serial sections with a thickness of 5 µm were cut from each sample. The sections were deparaffinized and stained with periodic acid Schiff reagent to highlight mucous and goblet cell secretion products stored in the cells. Goblet cells that were positively stained in the conjunctiva were counted. The results are presented as the average number of goblet cells per millimeter, acquired under a microscope (Leica DM750; Leica Microsystems).

### Transcriptome Sequencing

The process of obtaining RNA samples involved the use of TRIzol to extract total RNA following the instructions provided by the manufacturer. The RNA concentration and integrity were evaluated via a Bioanalyzer 2100 along with an RNA 6000 Nano LabChip Kit (Agilent, CA, USA; 5067-1511). cDNA libraries were sequenced on the Illumina NovaSeq 6000 platform, which produced paired-end sequences of 150 bp; this step aimed at achieving high-quality clean sequences by filtering out low-quality sequences through Cutadapt76 (v1.9) and verifying sequence quality via FastQC (v0.11.9). The sequences were mapped to the human reference genome (UCSC hg38) via HISAT2 77 (v2.0.4). StringTie 78 (v1.3.4d) then assembled transcriptomes for each sample, and gffcompare (v0.9.8) was used to compare the merged transcriptomes from all samples, whereby StringTie estimated the expression of all transcripts and generated count tables; subsequently, differential expression analysis between the two groups was conducted with the DESeq2 R package79 (v1.20.0).

### Statistical Analysis

The ggpubr package (v0.5.0, https://CRAN.R-project.org/package=ggpubr) was used to investigate differences between groups. The Kolmogorov-Smirnov and Shapiro-Wilk normality tests were used to assess the adherence of the data to a normal distribution. For datasets conforming to a normal distribution, parametric t tests were utilized for the analysis of two independent samples. Conversely, the Mann‒Whitney U test was applied to datasets that did not follow a normal distribution. For multiple sample groups adhering to a normal distribution, one-way ANOVA was conducted for pairwise comparisons, whereas the Kruskal‒Wallis test was selected for groups not exhibiting normal distribution patterns. The data are presented as the means ± standard deviations, and a P value of less than 0.05 was considered statistically significant. All of the statistical analyses were performed using R v4.0.3, GraphPad Prism 9 (GraphPad, La Jolla, CA, USA), and SPSS v19.0 (SPSS, Chicago, IL, USA).

## Results

### IL-23 Upregulates Conjunctival γδT Cells in Murine Models

**Figure 1.**
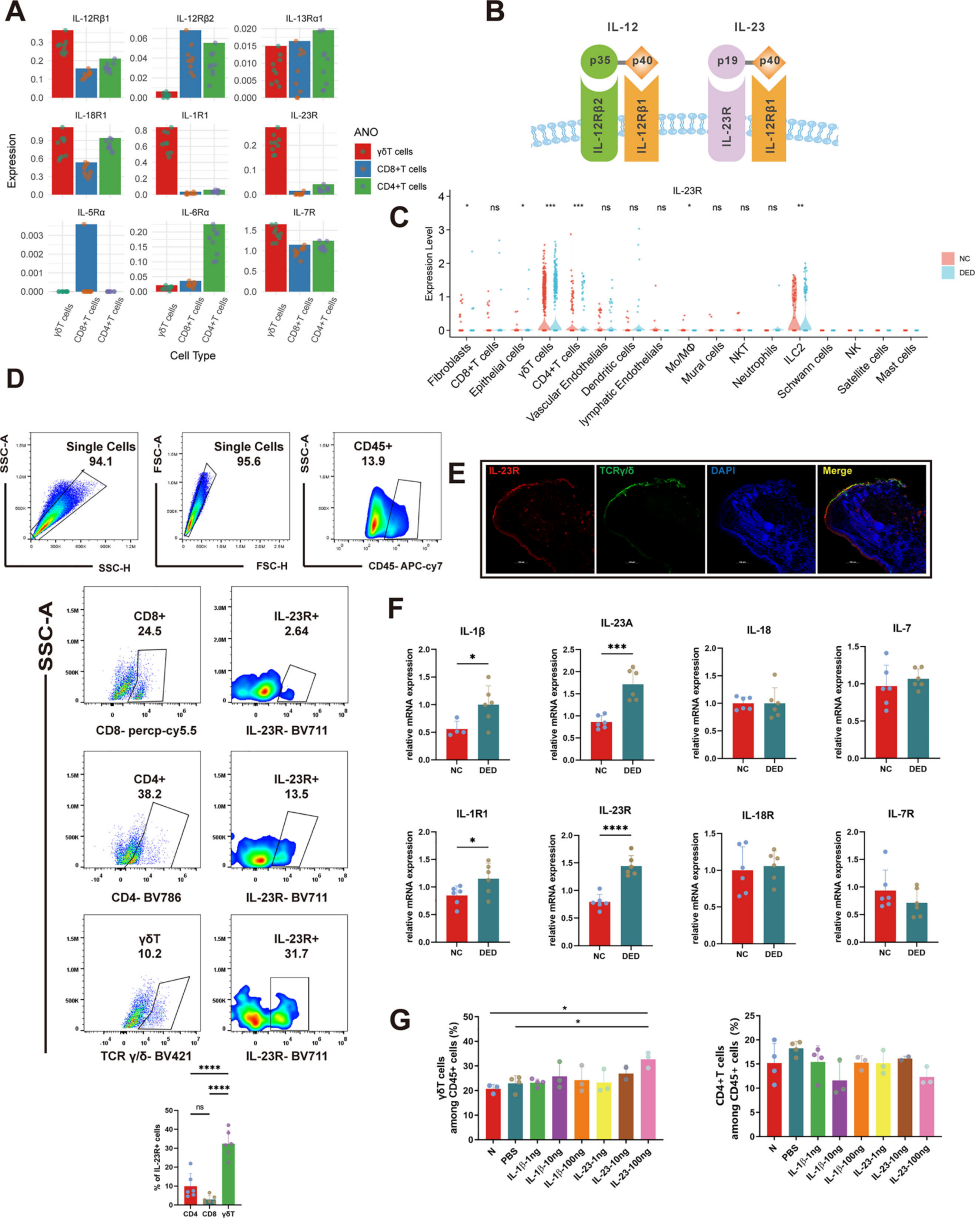
IL-23 upregulated conjunctival γδT cell expression in mice. (**A**) Receptors for inflammatory cytokines expressed by γδT cells were assessed via the gene set scoring method. (**B**) Molecular structures of IL-12R and IL-23R. (**C**) ScRNA-seq analysis of IL-23R expression levels in all conjunctival cells of mice examined by direct gene expression analysis. (**D**) Representative FCM schematic and statistical plot of IL-23R expression in lymphocyte subsets, including CD4+ cells, CD8+ cells, and γδT cells. (**E**) Immunofluorescence staining of IL-23R and TCR γ/δ to label γδT cells, with DAPI staining of the cell nuclei and a merged composite image. **(F)** Comparative analysis of the mRNA expression levels of IL-23R, IL-1R1, IL-7R, and IL-18R and the corresponding cytokines in the conjunctiva of DED and NC mice (*n* = 4–6, *P < 0.05, ***P < 0.001, ****P < 0.0001). (**G**) Proportion of γδT cells in CD45+ populations in the murine conjunctiva after subconjunctival injection of varying dosages of IL-1β and IL-23 (*n* = 2–4, *P < 0.05).

Conjunctival γδT cell activation is an essential response that mediates inflammation in murine DED.^23^ The conjunctival epithelial cells of the mice were harvested after desiccation in an ICES chamber. ScRNA-seq analysis revealed that increases in the expression of IL-23R and IL-12Rβ1, which are upstream mediators of γδT activation. However, only marginal expression of IL-12Rβ2 was detected. IL-1R1, IL-7R, and IL-18R expression levels were elevated, although these markers were not exclusive to γδT cells. Similar nonselective increases were observed in CD4+ and CD8+ T cells (Figs. 1A, 1B). IL-23R was highly expressed in murine conjunctival cells. Besides, the expression levels of IL-23R was significantly higher in DED versus control animals (Fig. 1C). Additionally, FCM was employed to assess IL-23R expression in lymphocyte subsets, including CD4+ cells, CD8+ cells and γδT cells. A high level of IL-23R expression was evident on γδT cells from WT mice. IL-23R expression was evident in the γδT population (32.42% ± 7.10%), which was significantly greater than that in the other lymphocyte subtypes (Fig. 1D). Furthermore, double immunofluorescence staining of conjunctival slices from WT mice, which targeted both IL-23R and TCR γ/δ, revealed substantial colocalization of these markers, suggesting a marked interaction between IL-23R and γδT cells (Fig. 1E).

We assessed the mRNA expression levels of IL-23R, IL-1R1, IL-7R, and IL-18R and the corresponding cytokines in the conjunctiva of DED and NC mice via RT‒qPCR. Both the IL-23R and IL-23 expression levels were elevated after DED induction, and the IL-1β expression level was also increased (Fig. 1F). We then administered different doses of the IL-23 and IL-1β proteins subconjunctivally and performed FCM to examine the proportion of γδT cells. Notably, an injection of 100 µg of IL-23 significantly increased the proportion of γδT cells (*P* < 0.05), whereas IL-1β had no statistically significant effect (Fig. 1G). These results indicate that IL-23 is a significant activator of γδT cells, whereas IL-1β lacks such a function. Collectively, these findings revealed that IL-23R is a specific marker for conjunctival γδT cells and validated the hypothesis that IL-23 is a primary up-regulator of γδT cells.

### IL-23 Depletion Suppresses Inflammatory Upregulation of γδT Cells

**Figure 2.**
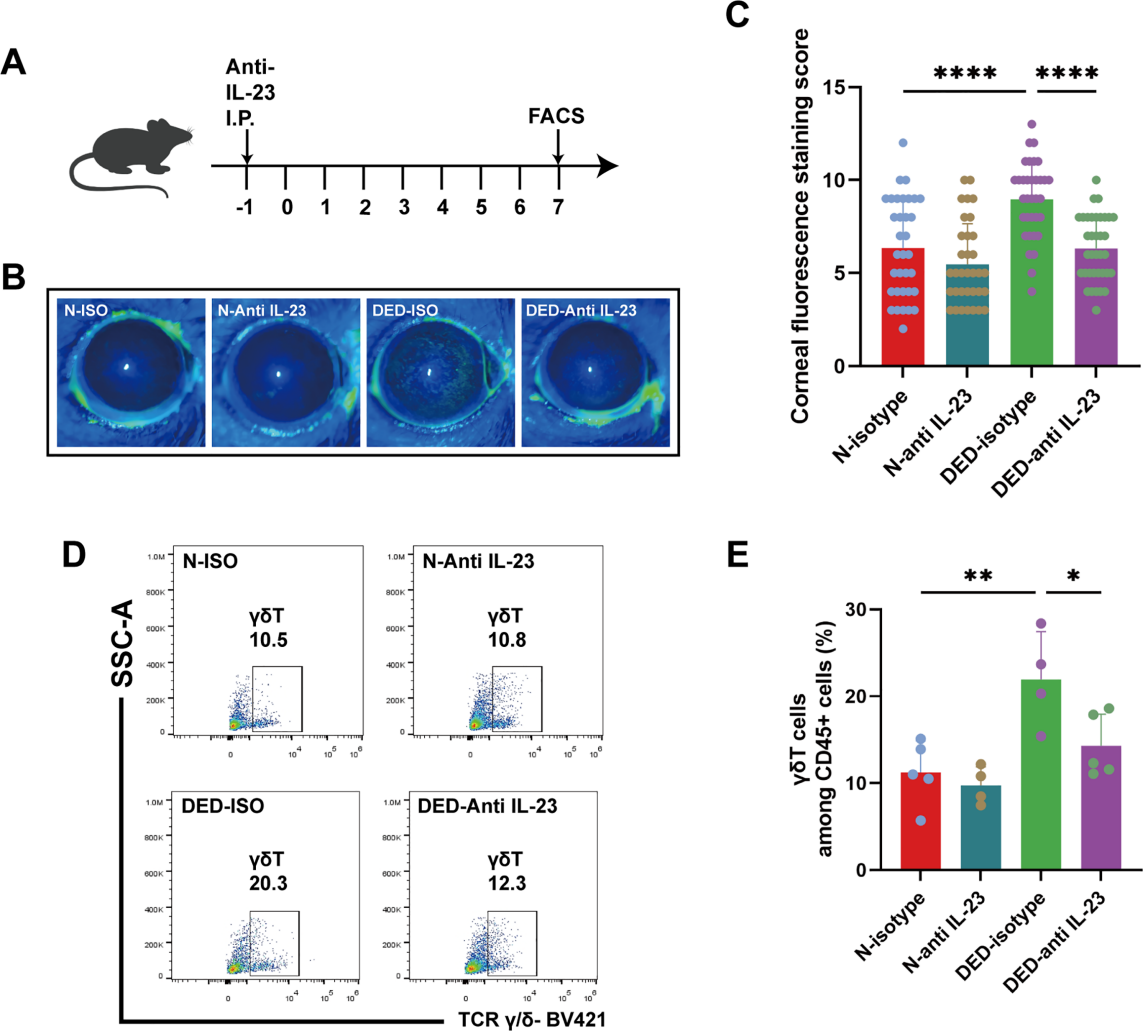
Blockade of IL-23 in mice via intraperitoneal injection of IL-23-neutralizing antibodies led to a reduced number of gdT cells and alleviated DED. (**A**) Timeline flowchart illustrating intraperitoneal anti-IL23 injection, DED modeling, and sampling for FCM analysis. (**B**, **C**) Corneal sodium fluorescein staining in the four groups of mice and corresponding statistical analysis (*n* = 34–36, ****P < 0.0001). (**D**, **E**) Flow schematic and statistical plot of conjunctival cells in mice post injection with IL-23-neutralizing antibodies (*n* = 4–5, *P < 0.05, **P < 0.01).

As IL-23 is a key modulator of γδT cell function, we identified its role in mediating inflammation in the ICES mouse model. One day before DED induction, we intraperitoneally administered anti-IL-23 neutralizing antibodies to the mice. On day 7 post DED induction, fluorescein sodium staining was used to evaluate the integrity of the ocular surface, and FCM was used to evaluate the conjunctival tissue samples (Fig. 2A). The fluorescein sodium staining scores of the DED mice treated with the anti-IL-23 neutralizing antibodies were significantly lower (6.33 ± 1.76) than those of their counterparts receiving the isotype control (8.97 ± 2.05) (P < 0.0001). This difference suggested that IL-23 attenuation ameliorates ocular surface disruption in DED. Furthermore, in the normal control group, the isotype control staining score was 6.35 ± 2.64, whereas in the group injected with the IL-23 neutralizing antibody, it was 5.47 ± 2.19, indicating a marginal decrease but not a statistically significant decline in the latter group (Fig. 2B, 2C and Supplementary Figure S2). FCM analysis revealed variations in γδT cell prevalence among the CD45+ cells in the conjunctiva. Consistent with the ocular surface findings, the γδT cell fraction in DED mice decreased to 14.30% ± 3.64% after IL-23 neutralization, whereas it was 21.95% ± 5.49% in the isotype control group. In normal control mice, the γδT cell fraction was 10.65% ± 2.77% in the group subjected to IL-23 neutralization, which was slightly lower than the 11.24% ± 3.65% in the isotype control group, but the difference was not statistically significant (Figs. 2D, 2E). We previously reported that γδT cells are the main cells that secrete IL-17A in the conjunctiva, which exacerbates dry eye symptoms.^23^ Therefore IL-23 is an upstream regulator of increased γδT activity that augments inflammation in DED. This interplay between IL-23 expression and γδT activity contributes to the exacerbation of DED pathogenesis.

### IL-23 Upregulates Proinflammatory Responses through γδT Cells

**Figure 3.**
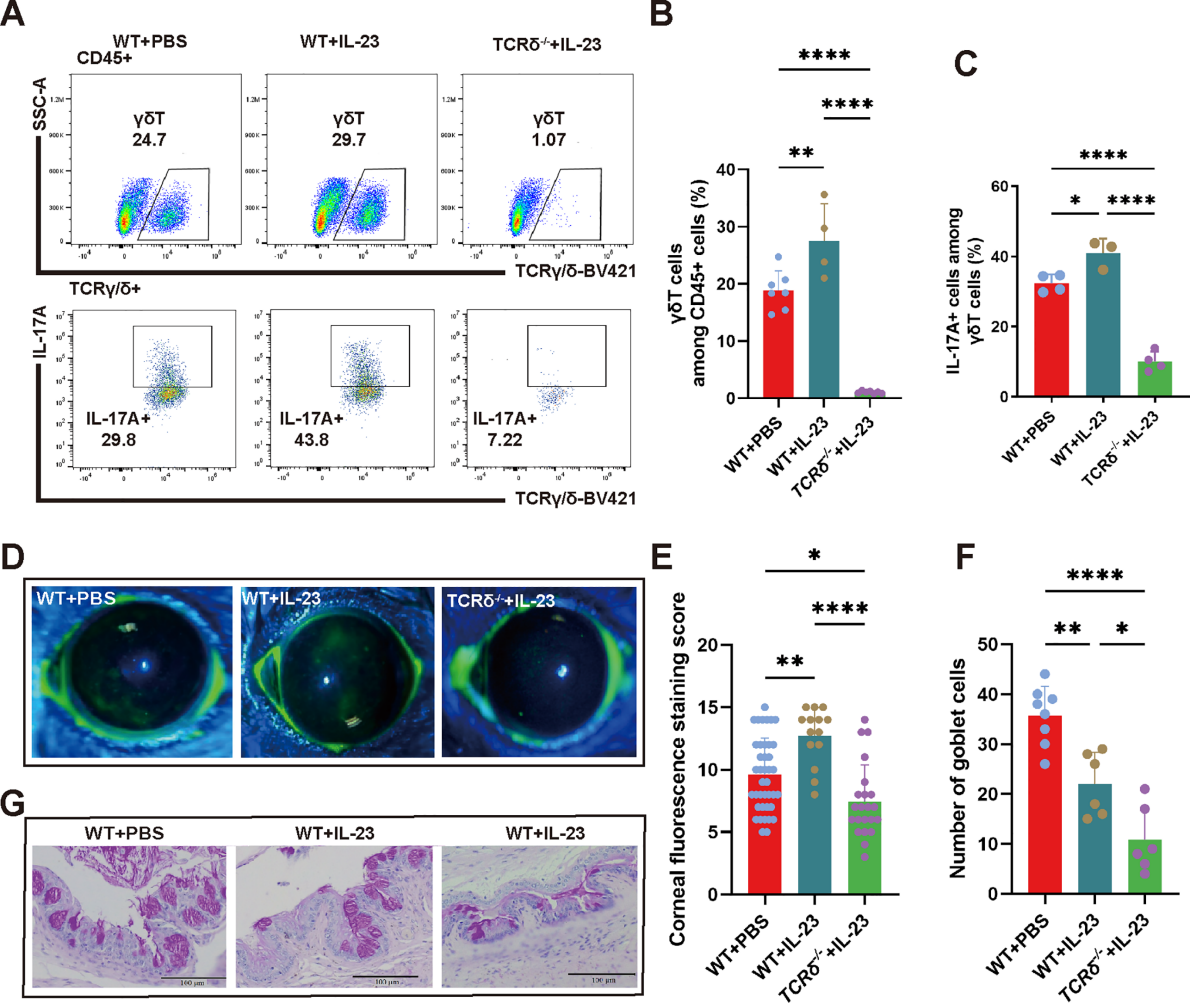
Analysis of the effects of IL-23 treatment on gdT cells and DED in WT and TCR*δ*^–/–^ mice. (**A**) Schematic γδT cell and IL-17A FCM gates in the three groups. (**B**, **C**) Statistical plots of the percentages of γδT- and IL-17A-expressing cells (n = 4–9, **P < 0.01, ***P < 0.001, ****P < 0.0001). (**D**, **E**) Evaluation of ocular surface integrity via sodium fluorescein staining: schematic and statistical analysis (*n* = 14–40, *P < 0.05, **P < 0.01, ****P < 0.0001). (**F**, **G**) Goblet cell visualization with PAS staining: schematic representation of the sodium fluorescein staining in mice and its statistical plot (*n* = 6–8, *P < 0.05, **P < 0.01, ****P < 0.0001).

To determine the functional role of IL-23 expression in conjunctival cells, the effect of subconjunctival injection of IL-23 was determined. Subsequent FCM analysis of the conjunctiva was used to assess the frequency of γδT cells and their IL-17A expression levels (Fig. 3A). A significant increase in the IL-17A expression level of γδT cells occurred after IL-23 injection (*P* < 0.05). In contrast, in IL-23-injected TCR*δ*^–/–^ knockout mice, both the proportion of γδT cells and their IL-17A expression levels remained markedly low (Figs. 3B, 3C).

In the PBS-injected WT control group, the fluorescein sodium staining score was 9.60%±2.92. Upon the administration of IL-23, this score significantly increased in WT mice. In contrast, the fluorescein scores of the IL-23-injected TCR*δ*^–/–^ knockout mice remained lower than those of the WT mice treated with PBS (Figs. 3D, 3E). Moreover, conjunctival PAS staining of goblet cells in the mice followed a similar trend, but the cell count in the TCR*δ*^–/–^ group injected with IL-23 was notably lower, likely because the deficiency of γδT cells affected the goblet cell density (Fig. 3F, 3G). Overall, these results indicate that IL-23 administration increases ocular surface DED symptomology in WT mice but that this procedure is less effective in TCR*δ*^–/–^ mice. This pattern underscores the critical role that γδT cells play in mediating the inflammatory responses mediated by increases in IL-23 within the conjunctiva.

### Unraveling the IL-23/γδT Signaling Pathway Axis in Human Dry Eye Disease

**Figure 4.**
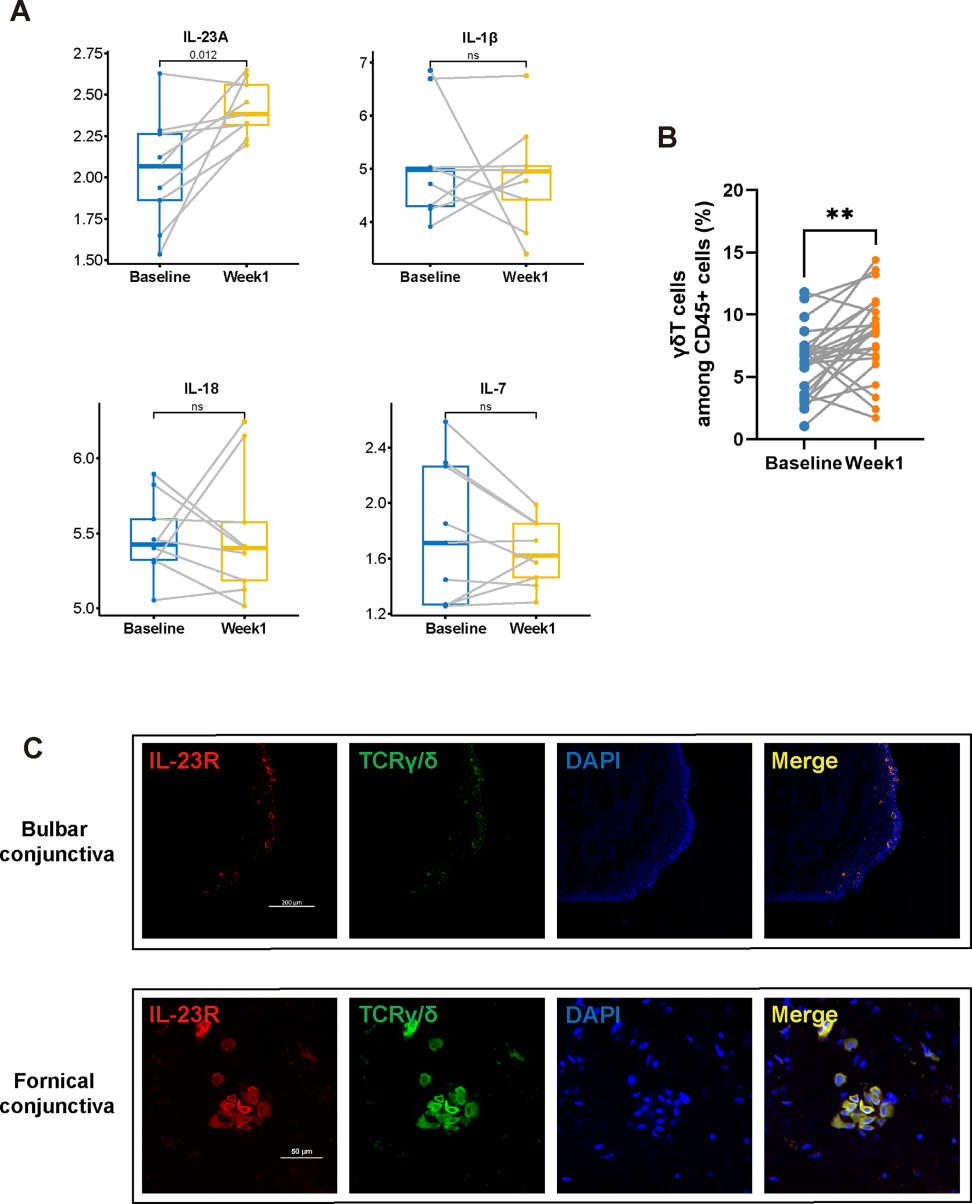
The correlation between the induction of IL-23 and the increase in γδT cell numbers on the human ocular surface. (**A**) RNA-Seq analysis of IL-1, IL-7, IL-18, and IL-23 in VDT users (*n* = 9, *P < 0.05). (**B**) FCM detection of the proportion of γδT cells among CD45+ cells in VDT users (*n* = 25, **P < 0.01). (**C**) Immunofluorescence double-staining of IL-23R and TCR γ/δ, which represent γδT cells, with DAPI staining for cell nuclei and a composite merged image.

We determined whether there is a relationship between the IL-23/γδT signaling pathway axis in mice and humans. Participants were recruited from the general population through advertisements, and swabs were used to obtain ocular specimens. A total of 26 participants were enrolled in the study, with an average age of 21.15 ± 2.23 years. Approximately 61.54% of the participants were female. All of the subjects were required to use VDT for more than 8 hours per day during the first week, followed by specimen collection at the end of the week. The VDT usage times at baseline and week 1 were 2.55 ± 0.46 hours and 11.17 ± 2.45 hours, respectively. After one week of use with VDT, the participants exhibited a statistically significant reduction in the tear-film breakup time (*P* < 0.05), whereas the corneal fluorescein staining and ocular surface disease index scores did not significantly change (Table). Ocular samples were subjected to FCM and RNA sequencing (RNA-seq) analysis. We specifically focused on IL-1β, IL-7, IL-18, and IL-23, as the scRNA-seq data revealed that they all increased in conjunctival γδT cells in the ICES model. Notably, IL-23A exhibited the highest expression increase compared to its baseline level (Fig. 4A). Concurrently, the proportion of γδT cells increased to 7.10% ± 5.34% after one week (Fig. 4B). These data underscore the key role of IL-23A in controlling the expression of proinflammatory cytokines that underlie inflammatory symptoms in human dry eye disease by γδT cells.

**Table.** Clinical Characteristics of Participants at Baseline and Week One

Characteristics	Baseline	Week 1	*P* Value
TBUT ± SD	6.35 ± 1.91	5.17 ± 2.24	0.002
CFS ± SD	0.35 ± 0.79	0.42 ± 0.94	0.833
OSDI ± SD	6.35 ± 1.93	5.17 ± 2.26	0.742

CFS, corneal fluorescein staining; OSDI, ocular surface disease index; SD, standard deviation; TBUT, tear-film breakup time (grade estimated by the Oxford scheme).

We used double immunofluorescence staining of human conjunctival sections, including bulbar and fornical conjunctiva sections, and targeted assessment of the IL-23R and TCR γ/δ expression levels. The results revealed significant colocalization of these markers (Fig. 4C), confirming the presence of the IL-23/γδT signaling pathway axis on the human ocular surface.

## Discussion

We show here that IL-23 upregulation contributes to mediating the inflammatory response that is a characteristic of DED in both humans and mice. This effect is accompanied by an increase in γδT cell density, which is proportional to the disruption of ocular surface integrity. The signaling pathway axis was identified by using optimized next-generation DNA sequencing technologies. Implementation of this technology enables detection of the effects of changes in the microenvironment on DNA sequence variations that underlie functional differences resulting in DED.^55^^–^^57^ Furthermore, RNA sequence analysis probes for the expression of marker genes that are characteristic of different cell types.^58^^–^^60^ This approach elucidates the interactions between different cell types that induce different responses.^61^^–^^63^ Our use of this technology revealed that IL-23R is a highly expressed cognate membrane receptor through which IL-23 interacts with γδT cells to disrupt ocular surface integrity and inflammation in DED. The different techniques used to achieve this conclusion are shown in Figure 1.

SCRNA-seq data analysis showed that along with upregulation of IL-23R, the expression levels of IL-12R1, IL-7R, IL-18R, and IL-1R1 also increased in γδT cells. The analysis also revealed that IL-23 binds to the IL-23R and IL-12R1 complex on the surface of γδT cells to activate downstream inflammatory signaling pathways.^64^ Notably, the receptor for IL-12 also forms a complex composed of IL-12R1 and IL-12R2.^65^ The increases in the expression levels of IL-12R1 and IL-23R on γδT cells, which are associated with decreases in IL-12R2 expression, indicate that conjunctival γδT cells predominantly respond to IL-23 rather than to IL-12 (Fig. 1A, B). RT‒qPCR analyses confirmed this conclusion. IL-7 expression varies across lymphoid cells^66^^–^^68^ and likely promotes the proliferation of all cells of the lymphoid lineage^69^^,^^70^; thus it does not mediate inflammation in DED (Fig. 1F). IL-18 was initially described as a cytokine capable of inducing mature Th1 cells to produce IFN-γ independently of IL-12.^71^ However, more recent findings indicate that in the absence of IL-12, exogenous IL-18 cannot drive the Th1 differentiation of naive cells.^72^^,^^73^ Furthermore, only in conjunction with IL-12 can mature Th1 cells be induced to upregulate IFN-γ expression.^73^ A study by Lino et al.^47^ revealed that the enhancement of the Th-1 response and the production of IFN-γ depend on an interaction between IL-12 and IL-18 to promote the immune response in NKT cells. However, the low expression level of the IL-12R complex on γδT cells implies that IL-18 may not play a significant role in controlling the functional status of conjunctival γδT cells in mice (Fig. 1A). Our study demonstrated that injection of IL-23 induced an increase in the proportion of γδT cells as a consequence of the interaction of IL-23 with IL-23R on γδT cells within the conjunctiva. In contrast, IL-23R expression is much lower in other cell types, which renders the injected IL-23 ineffective. Conversely, despite its upregulated expression on γδT cells, IL-1R1 is also highly expressed in other conjunctival cells, such as vascular endothelial cells and fibroblasts. Thus these results lead to the dissociation of IL-1β from the inflammatory pathway related to the induction of DED in γδT cells. Hence, we propose that IL-23 is the primary upstream regulator of the inflammatory response of mouse conjunctival γδT cells in DED.

IL-23 is a heterodimeric cytokine that consists of the IL-23 alpha subunit paired with the IL-12p40 subunit.^74^ This IL-12p40 subunit, also known as the IL-12 subunit beta, plays a multifunctional role. It associates with IL-23p19 to assemble with IL-23 and pairs with IL-12A, thereby forming IL-12.^74^ To investigate the specific function of IL-23, a targeted approach in which an IL-23 neutralizing antibody was intraperitoneally injected into mice was used. This antibody specifically interacts with the p19 subunit of mouse IL-23, which selectively eliminates IL-23 bioactivity. After its administration, the proportion of γδT cells did not increase, and ocular surface symptoms were unchanged (Fig. 2). In WT mice, the induction of IL-23 expression led to a notable increase in the proportion of γδT cells, along with intensified inflammation at the ocular surface. This finding underscores the pivotal role of IL-23 in modulating immune responses, particularly through its interaction with γδT cells, leading to the secretion of IL-17A, which then exacerbates ocular surface symptoms. In contrast, in γδT cell-deficient TCR*δ*^–/–^ mice, the injection of IL-23 did not lead to an increase in the sodium fluorescein staining score. This outcome suggests that in the absence of γδT cells, other immune cells, such as CD4+ and CD8+ T cells, contribute less to inflammation at the ocular surface (Fig. 3). Therefore γδT cells are required for mediating IL-23-driven inflammatory responses at the ocular surface. Unexpectedly, the goblet cell density in the conjunctiva of IL-23-injected TCR*δ*^–/–^ mice was significantly lower than that in the conjunctiva of WT mice. Kober et al.^75^ reported that, compared with those in WT mice, the number of goblet cells and crypt length in both the small intestine and colon were lower in TCR*δ*^–/–^ mice. The disruption of mucosal homeostasis stems from the pronounced conjunctival goblet cell deficiency that occurs in TCR*δ*^–/–^mice. Our comprehensive approach revealed that IL-23 upregulation increases IL-17 expression by γδT cells. This response contributes to increases in ocular surface inflammation during DED. These findings suggest that the development of IL-23R blockers may provide an approach to suppress inflammation in the clinical setting to treat DED.

We probed for upstream inflammatory factors that affect DED progression by analyzing differences between baseline and one-week ocular surface cell RNA-seq data. The results indicated that among all of the investigated inflammatory factors, only the expression level of IL-23 increased after one week. This response suggests that IL-23 is an upstream regulatory factor of γδT cells on the human ocular surface and that its interaction with IL23R contributes to the inflammatory response underlying dry eye disease development. Additionally, the proportion of γδT cells increased after one week, but the proportion of γδT cells in the human eye differed significantly from that in the mouse conjunctiva. This discrepancy may be due to differences in species expression and collection methods. In mice, the entire globe, eyelid conjunctiva, and fornix conjunctiva are collected, whereas ocular swabs can only collect superficial lymphocytes in humans. Immunofluorescence was used to detect clusters of γδT cells in the human conjunctiva, which form lymphoid follicles similar to conjunctival-associated lymphoid tissue, as described in several papers.^76^^,^^77^ These immune follicles develop after birth and contain all of the necessary cells for inducing an immune response, including bone marrow-derived APCs, B cells, and T cells, with up to 50% being γδT cells,^77^ highlighting the importance of γδT cells in conjunctival immunity.

One of the limitations of our study is that it is focused primarily on the contribution of conjunctival γδT cells to mediating inflammatory responses during dry eye disease. We identified γδT cells in the human corneal limbus, indicating the need for further exploration into the early activation of γδT cells in the cornea and their potential interactions with corneal nerves. Moreover, our investigation did not address the migratory pathways of γδT cells from the ocular surface to the cervical lymph nodes or their potential associations with systemic immunity. These limitations highlight areas for future study. Their investigation is expected to reveal the nuanced roles and mechanisms of γδT cells within the wider immune landscape.

In summary, we provide definitive evidence that IL-23 is an upstream regulatory factor of γδT cells that plays a crucial role in controlling ocular surface inflammation in DED. The inflammatory IL-23/γδT signaling axis is a key component of the immune-inflammatory mechanism of DED in both mouse models and humans. This function is dependent on an interaction between IL-23 and its cognate receptor, which is expressed primarily by conjunctival γδT cells. Accordingly, these findings identify potential targets for inhibiting the IL-23/γδT signaling pathway axis and improving the therapeutic management of DED in a clinical setting.

## Supplementary Material

Supplement 1
