# Supplementary material for: IL-23 Promotes γδT Cell Activity in Dry Eye Disease Progression
Source: Invest Ophthalmol Vis Sci. 2025 Feb 4;66(2):10. doi: 10.1167/iovs.66.2.10 (PMC11801388; doi:10.1167/iovs.66.2.10)
Supplement: Supplement 1 [file iovs-66-2-10_s001.pdf]

Supplementary Information

IL-23 Promotes γδT Cell Activity in Dry Eye Disease Progression

Yanxiao Li [#, 1,2], Zan Luo [#, 1,2], Zihao Liu [1,2], Xinhao Zhu [1,2], Peter S Reinach [1,2], Ling Li ✉ [3] Wei Chen ✉ [1, 2,3]

[1] State Key Laboratory of Ophthalmology, Optometry and Visual Science, Eye Hospital, Wenzhou Medical University, Wenzhou, 325027, China

[2] National Clinical Research Center for Ocular Diseases, Eye Hospital, Wenzhou Medical University, Wenzhou, 325027, China.

[3] Ningbo Eye Institute, Ningbo Eye Hospital, Wenzhou Medical University, Ningbo 315040, China.

[#] These authors contributed equally: Yanxiao Li, Zan Luo.

✉ Correspondence: Wei Chen, Wenzhou Medical University, 270 West Xueyuan Road, Wenzhou, Zhejiang 325027, China. e-mail: chenweimd@wmu.edu.cn

Ling Li, Ningbo Eye Hospital, Wenzhou Medical University, 599 North Mingcheng Road, Ningbo, Zhejiang 315040, China. e-mail: Liling498@126.com

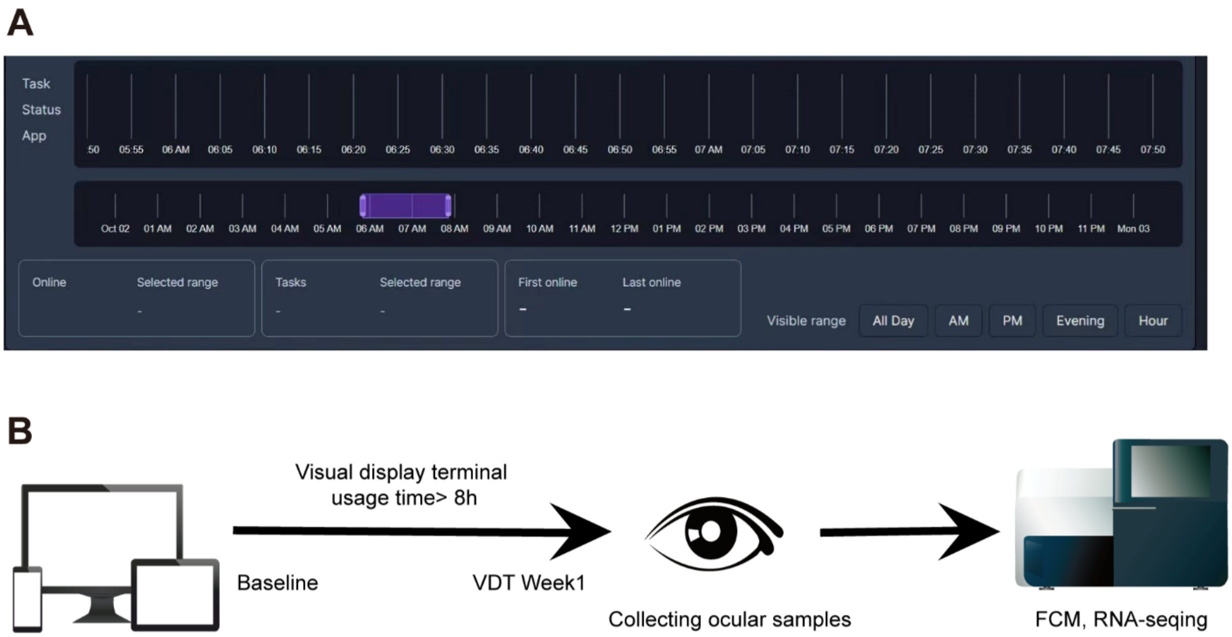

Figure S1. Details about clinical subject. (A) The example data of Tockler. (B) specific process of the clinical trial.

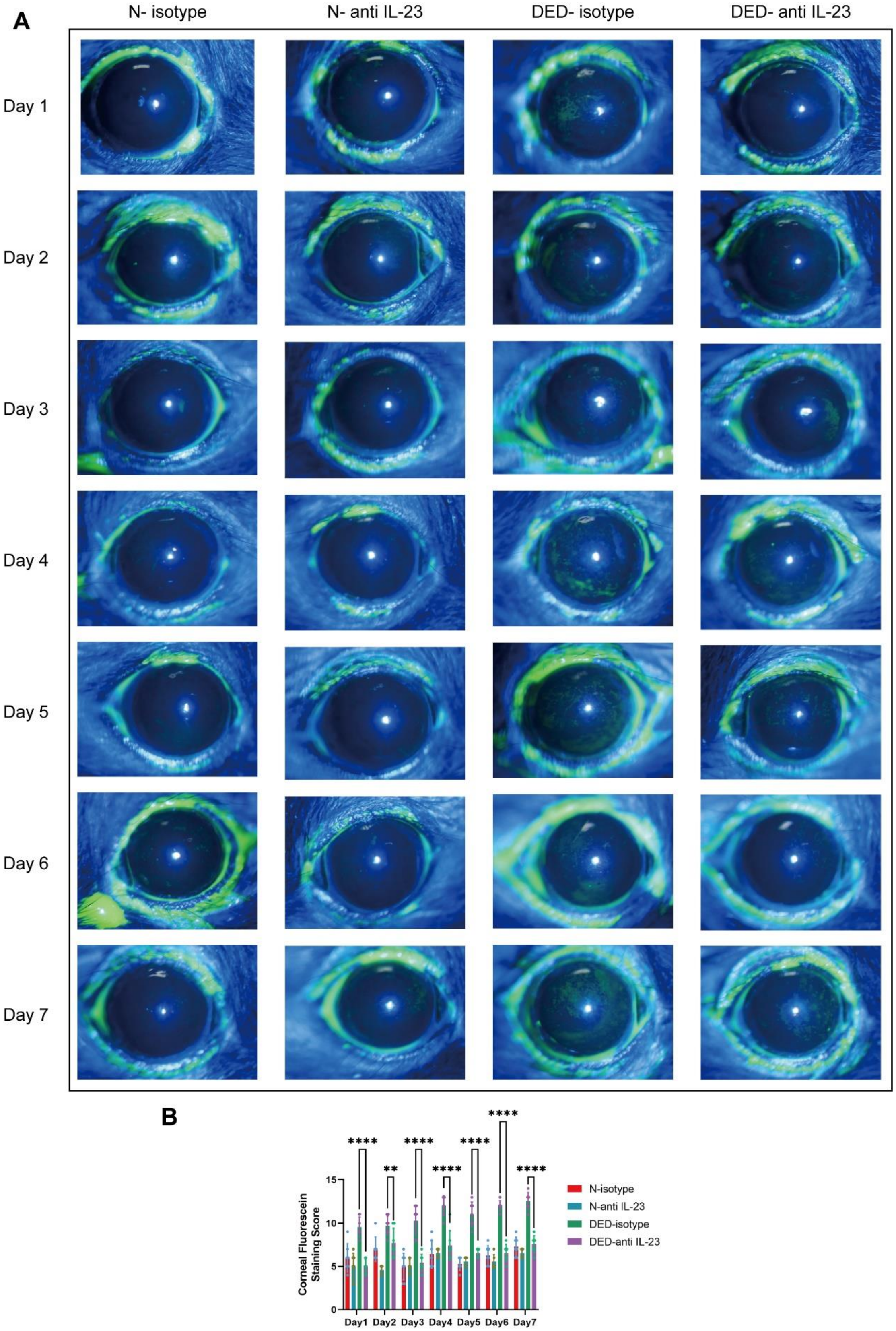

FigureS2. Corneal sodium fluorescein staining in the four groups. (A) Schematic of ocular surface sodium

fluorescein staining. (B) Statistical analysis of sodium fluorescein staining results (n=5-7, ****$p < 0.0001$)
